# Bone regeneration using a porcine bone substitute collagen composite *in vitro* and *in vivo*

**DOI:** 10.1038/s41598-018-19629-y

**Published:** 2018-01-17

**Authors:** Eisner Salamanca, Chia-Chen Hsu, Haw-Ming Huang, Nai-Chia Teng, Che-Tong Lin, Yu-Hwa Pan, Wei-Jen Chang

**Affiliations:** 1 School of Dentistry, College of Oral Medicine, Taipei Medical University, Taipei, Taiwan; 2 Dental Department, Taipei Medical University, Shuang-Ho hospital, Taipei, Taiwan; 3 Dental Department, Taipei Medical University Hospital, Taipei, Taiwan; 4 Department of General Dentistry, Chang Gung Memorial Hospital, Taipei, Taiwan; 5 Graduate Institute of Dental & Craniofacial Science, Chang Gung University, Taoyuan, Taiwan; 6 School of Dentistry, College of Medicine, China Medical University, Taichung, Taiwan

## Abstract

The biocharacteristics of xenogeneic grafts make them a possible substitute for autogenous bone grafts in dental bone graft procedures. This study aimed to develop a novel porcine graft with collagen capable of generating new bone in bone defects via osteoconduction over 8 weeks of healing and to compare it with a porcine graft. The porcine collagen graft was made to undergo a cell viability test (MTT) and alkaline phosphatase assay (ALP). The surgical procedure was performed in 20 male adult New Zealand white rabbits. Four calvarial critical-size defects of 6 mm in diameter were prepared in each rabbit. The upper left defect was filled with a porcine graft of 500–1000 μm, the upper right with a porcine collagen graft, the lower left with hydroxyapatite/beta-tricalcium phosphate and the lower right served as the control without any filling material. The rabbits were divided and sacrificed at 2, 4, 6 and 8 weeks after surgery. Histological and micro-CT scan results showed that the performance of the porcine collagen graft is superior for regenerating new bone. Porcine collagen graft showed cell viability and osteoblast-like cell differentiation *in vitro*. The results indicate that porcine collagen graft is a potential bone substitute for clinical application.

## Introduction

Bone graft materials are often used in dental treatments, such as infrabony defects, furcation defects, ridge augmentation, socket preservation, peri-implant defects and sinus augmentation^1^. For years, autologous bone grafts have been and are still considered the gold standard graft for these treatments because they produce bone by cellular proliferation from viable transplanted osteoblasts, by osteoconduction of cells along the graft’s surface or by osteoinduction of recruited mesenchymal cells. There is no risk of rejection with this technique, and it is highly reliable for bone regeneration treatments due to its low antigenicity^2^. However, it has some major disadvantages, including poor osseointegration and excessive resorption when the defect is >6–9 cm or when the surrounding tissues do not provide sufficient blood supply due to scarring, infection or irradiation^3^. Moreover, autologous tissue use requires the sacrifice of healthy tissue at the donor site, resulting in additional morbidity^4^. Intraoral sites do not provide enough graft volume; extraoral sites can provide a greater volume^5^ but carry increased costs for the patient. For dental surgeons, the process of harvesting healthy bone from intraoral or extraoral sites entails a slow and complicated learning curve, and postsurgical complications may occur during the learning period^6^.

In multiple guided bone regeneration (GBR) studies, large numbers of patients with different needs and requirements combined with autogenous bone limitations made it necessary for scientists and clinicians to search for alternative grafts, such as an allogenous graft, xenograft and allograft; all of these materials have shown different types of physicochemical characteristics and reliability in specific treatments^7–12^. A bovine bone graft is the most commonly used material of the xenograft biomaterials. The primary concern with its use is the possible iatrogenic transmission of prion-related diseases to patients treated with this product. Although the risk had declined due to appropriate preventive measures^1^, it was necessary to study another type of xenograft material with fewer disease transmission possibilities than the bovine graft^13^. The density of this xenograft is closer to that of the human bone^14^. The porcine graft has all of these characteristics and has exhibited features such as osteoinduction and osteoconduction in various studies in recent years^10,15,16^. Hence, it is a reliable biograft for GBR.

Type I collagen is another biomaterial that has proven to be useful in GBR treatments because it is a natural 3-dimensional (3D) structural component of tissues and the main constituent of bone tissue’s extracellular matrix. It is an excellent delivery system for growth factors and facilitates osteoblast and vessel migration and penetration, thus promoting angiogenesis and new bone formation^17^. Previous studies have shown that a mixture of type I collagen and sintered bovine bone form a 3D structure with better clot preservation and angiogenesis and demonstrate alveolar bone preservation and enhanced bone tissue engineering^17^. The addition of type I collagen to porcine bone substitute may preserve the clot, and type I collagen has the physical characteristics of a hard sponge in the porcine graft material and is easy to adapt its form to the shape of the defect after it is hydrated; its fibrillar structure provides a scaffold for cell ingrowth and regeneration, supporting angiogenesis and enhancing porcine graft regeneration characteristics during GBR treatments^18^.

Ultimately, animal systems are required to test the safety and efficacy of data acquired before transferring to humans^19^. Originally developed to simulate fracture nonunion in long bones, the calvarial critical-size defect in rodents and rabbits is perhaps the most widely used preclinical *in vivo* model for screening bone biomaterials^20^. In a previous study on rabbits with critical-size defects conducted in the same laboratory as the present study, porcine bone grafts were used in a similar manner as commercial hydroxyapatite/beta-tricalcium phosphate (HA/β-TCP) by regenerating bone formation through osteoconduction. However, new bone generation and particle manoeuvring during surgery with the porcine graft could be improved for future use in the dental clinic. Therefore, a novel composite for GBR treatments was developed by combining a porcine bone substitute with homogenous collagen and freeze-drying it.

This study aimed to develop a novel composite combining a porcine graft with collagen, to evaluate its characteristics *in vitro* using New Zealand rabbit calvarial critical-size defects and to assess its reliability as a bone graft biomaterial for new bone formation in future GBR treatments.

## Results

### Scanning Electron Microscope Examination

**Figure 1:**
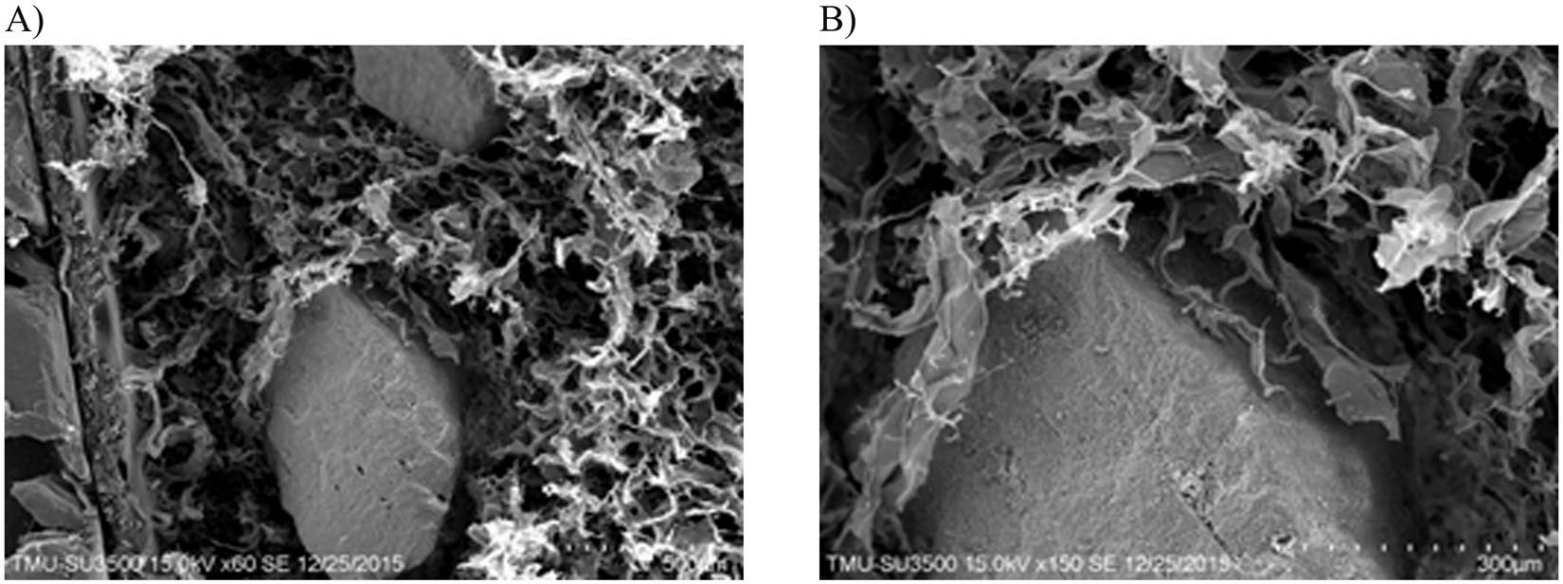
Porcine collagen SEM. The scanning electron microscope image shows the porcine bone substitute granules’ homogenous (Fig. 3A, ×60 magnification) integration within the collagen matrix (Fig. 3B, ×350 magnification).

Scanning electron microscope (SEM) examination showed porcine granules homogenously distributed within the collagen matrix (Fig. 1A). At a higher magnification, the collagen matrix presented a rough surface while surrounding and being in direct contact with porcine bone substitute particles (Fig. 1B).

### Energy Dispersive Spectrometry

Energy-dispersive spectrometry (EDS) analyses showed that the carbon (C) element had the atomic weight (62.17%), followed by oxygen (O) with 21.66%. Calcium (Ca) and phosphorus (P) were 7.54% and 4.58%, respectively, with a Ca/P ratio of 1.646.

### Cell Viability and Biocompatibility

**Figure 2:**
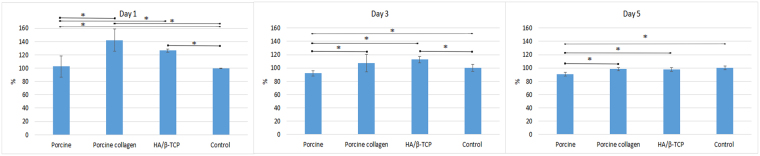
MTT assay. MTT assay of MG-63 cells at 1, 3 and 5 days. All the groups with graft materials were statistically significantly better than the control group at 1 day and showed viability during the 5 days of testing. Asterisks (*) indicate statistically significant differences (*P* < *0.05*).

The spectrophotometric methyl tetrazolium assay (MTT) assay results are presented in Fig. 2 and show that when the different graft biomaterials with MG-63 cells were cultured in the prepared media over 5 days, they were non-toxic and statistically significantly more viable than the control group at 1 day. At 3 days, only porcine collagen and HA/β-TCP were better than the control group, and all groups behaved similarly at 5 days *(P* < *0.05)* (Fig. 2).

### Alkaline Phosphatase Assay

**Figure 3:**
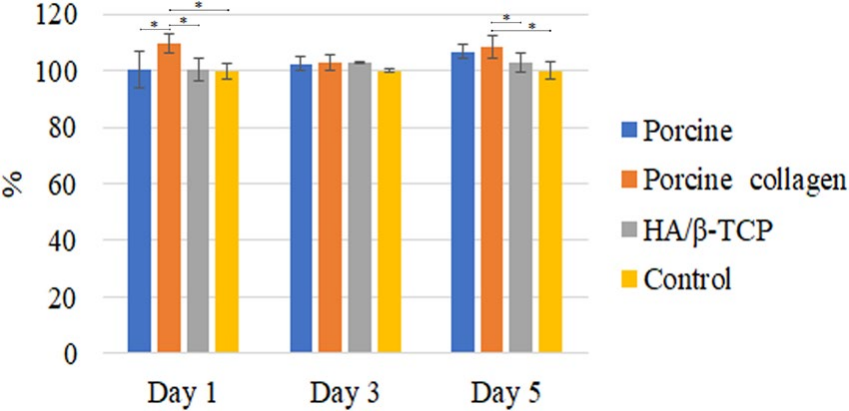
ALP test. Porcine collagen induced cells into osteoblast differentiation. Asterisks (*) indicate statistically significant differences (*P* < *0.05*).

**Figure 4:**
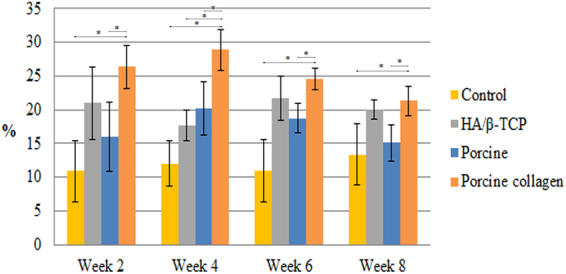
Micro-CT bone volume/tissue volume of new bone formation. New bone formation in relation to tissue volume in rabbit coronal defects. Asterisks (*) indicate statistically significant differences between each group and the porcine collagen group (*P* < *0.05*).

The alkaline phosphatase (ALP) assay showed that different graft materials increased the cells’ ALP activity in a time-dependent manner. The porcine graft and HA/β-TCP groups were slightly better than the control group at 1, 3 and 5 days, with no statistically significant differences. The porcine collagen graft group was always better than the other groups with only statistically significant differences over the other groups at day 1 and over the HA/β-TCP and control groups at day 5 (*P* < *0.05*) (Figs 3 and 4).

### Micro-CT Scanning

New bone formation at week 2 in the centre of the calvarial defects filled with HA/β-TCP, porcine graft and porcine collagen composite produced 21.0% ± 5.4%, 16.0% ± 5.1% and 26.4% ± 3.2%, respectively, being superior to the 10.9% ± 4.6% new bone formation in the control group. The porcine graft had a higher statistically significant difference (*P* < *0.05*) compared with the porcine graft and control groups (Table 1, Fig. 4).

**Table 1:** Micro-CT new bone formation.

**BV/TV (%)**	**Week 2**	**Week 4**	**Week 6**	**Week 8**
Control	10.9 ± 4.6	12.0 ± 3.4	11.0 ± 4.6	13.3 ± 4.6
HA/β-TCP	21.0 ± 5.4	17.7 ± 2.3	21.7 ± 3.3	20.0 ± 1.4
Porcine graft	16.0 ± 5.1	20.2 ± 3.9	18.8 ± 2.2	15.1 ± 2.6
Porcine collagen	26.4 ± 3.2	28.9 ± 3.0	24.5 ± 1.6	21.3 ± 2.2

Mean new bone formation ± standard deviation.

At week 4, the control group had the least amount of new bone formation (12.0% ± 3.4%) with a statistically significant difference compared with the other groups. HA/β-TCP had the second least amount of new bone formation (17.7% ± 2.3%). The porcine collagen group regenerated new bone (28.9% ± 3.0%) with statistically significantly higher values than those of the porcine graft (20.2% ± 3.9%) and other groups *(P* < *0.05)* (Table 1).

At week 6, the porcine collagen group had the most new bone formation (24.5% ± 1.6%), which was significantly more than those in the porcine graft (18.8% ± 2.2%), HA/β-TCP (21.7% ± 3%) and control groups (11% ± 4.6%). The porcine graft had a statistically significant difference (*P* < *0.05*) compared with the porcine graft and control groups (*P* < *0.05*) (Table 1).

At 8 weeks, the porcine collagen composite created more new bone (21.3% ± 2.2%) than the other groups, although the HA/β-TCP group (20.0% ± 1.4%) produced a similar amount. Similar to week 6, the porcine graft group (18.8% ± 2.2%), followed by the control group (11% ± 4.6%), generated the least amount of new bone. The defect closure at 8 weeks presented the same results as those in week 4. Porcine graft generated statistically significantly more bone (*P* < *0.05*) than the porcine graft and control groups (Table 1).

### Histology and Histomorphometric Analysis

Histological results revealed significant differences between the defects filled with graft materials and the control group. At all time points, some HA/β-TCP and porcine graft particle displacements were observed due to the lack of a barrier membrane. All the defects filled with the different materials managed to hold the critical-size defect spaces.

**Figure 5:**
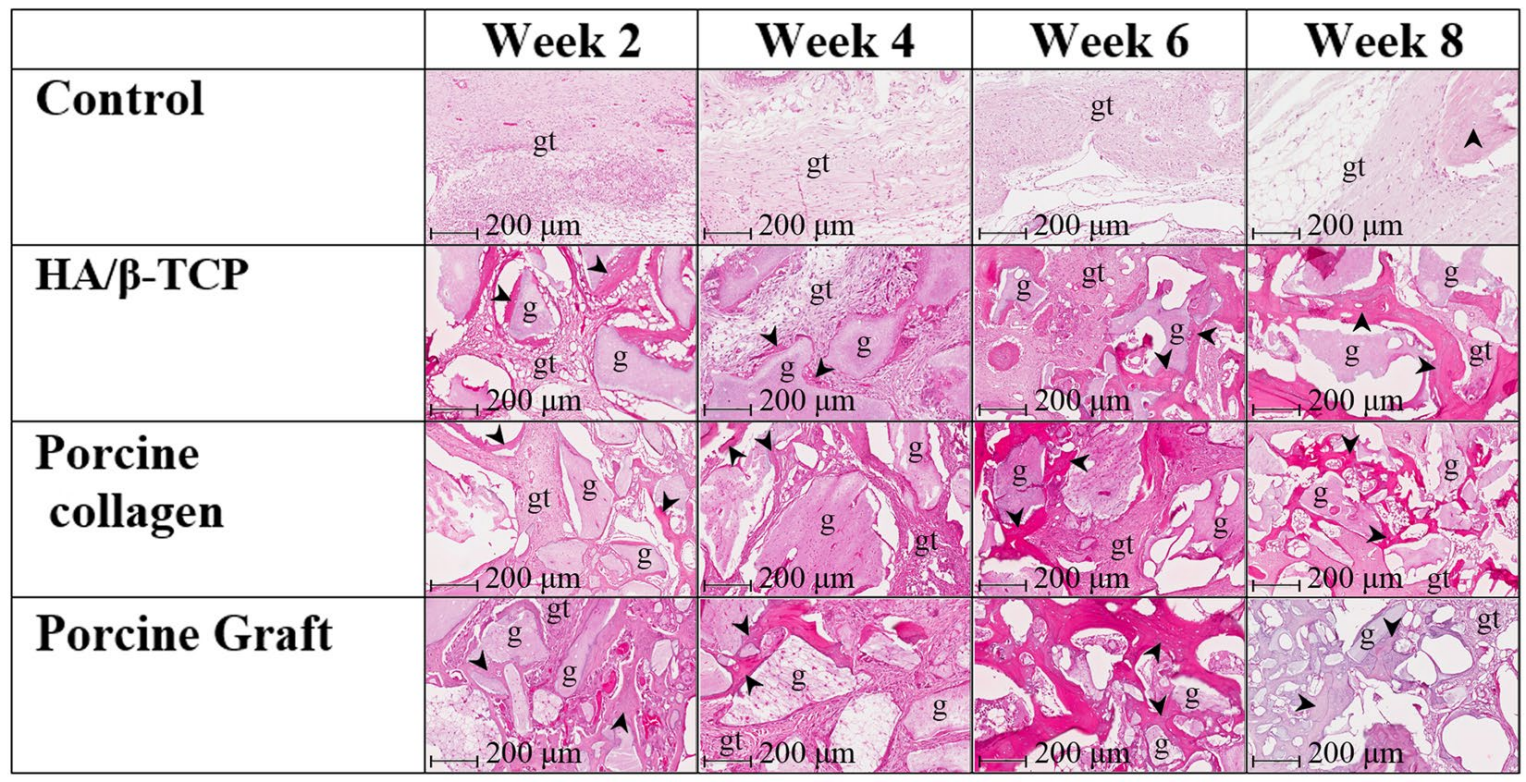
Sagittal view of the histology of cortical defects. Pictures of a mid-sagittal section that were previously taken from the cortical defect’s centre. Pictures show the healing process from 2 to 8 weeks in the different groups. gt: granulation tissue, g: graft particles, black arrowheads: new bone.

**Figure 6:**
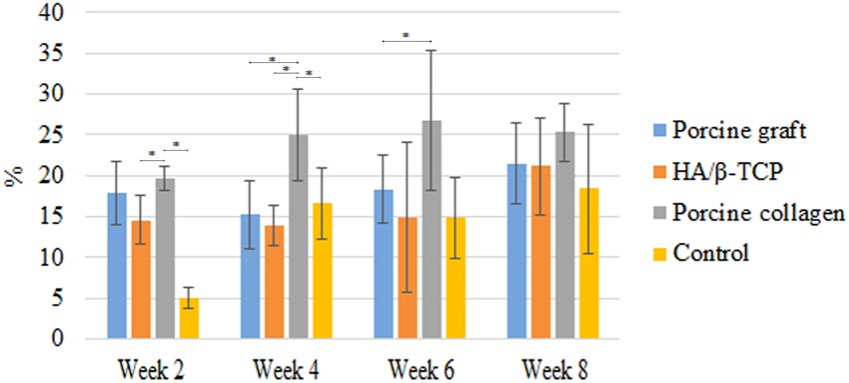
Histology of bone area/tissue area of new bone formation. New bone formation in relation to tissue area in rabbit coronal defects. Asterisks (*) indicate statistically significant differences (*P* < *0.05*).

During the second week, it was possible to observe inflammatory cells and predominantly woven bone surrounded by osteoblasts in all the groups. Close contact between the bone and graft was significantly less in the groups filled with graft materials. The greatest concentrations of immature bone were in the defects’ borders, with graft particles working as scaffolds while they were embedded in newly formed bone, which occasionally bridged the particles with branches of woven bone. The control group had the lowest bone formation at 5.06% ± 1.25%, whereas the porcine collagen group had the highest bone formation at 19.65% ± 1.46%. Porcine graft and HA/β-TCP groups had similar bone regeneration at 17.92% ± 3.88% and 14.56% ± 2.97%, respectively. The porcine graft group had a statistically significant difference (*P* < *0.05*) compared with the HA/β-TCP and control groups (Figs 5 and 6).

At 4 weeks, the porcine collagen group had the statistically significant highest difference in bone formation with 25.06% ± 5.62% (*P* < *0.05)*. Porcine graft, controls and HA/β-TCP groups had similar bone regenerations at 15.22% ± 4.22%, 16.60% ± 4.33% and 13.99% ± 2.46%, respectively. The graft biomaterials were already well integrated into the host tissue, forming an irregular surface boundary caused by the gradual degradation of the material. The interface developing between the graft particles and the surrounding tissue corresponded in structure and morphology to new bone tissue. New bone maturation processes started to be visible with the calcification of osteocytes in all groups (Figs 5 and 6).

At week 6, we observed significant new bone formation on the surface of some graft particles. Samples exhibited histological patterns of biomaterial granules with osteogenic activity predominant on the defect’s walls, in which small spaces were observed between graft material granules and the new bone. Just as in the previous weeks, the porcine collagen group generated more new bone (26.82% ± 8.59%), having only a statistically significant difference compared with the porcine graft group (*P* < *0.05*) with 18.34% ± 4.17% new bone. The control and HA/β-TCP groups produced the least new bone with 14.95% ± 4.94% and 14.89% ± 9.18%, respectively (Figs 5 and 6).

Histology slides demonstrated resorption of the grafts’ biomaterials at week 8. The porcine graft (21.48% ± 4.97%) and HA/β-TCP groups (21.13% ± 5.84%) had similar new bone formations; these results were close to the 25.30 ± 3.51% new bone formation generated by the porcine collagen group, whereas control defects had the least new bone formation (18.37% ± 7.93%). The presence of mature bone formation was differentiated from that in the graft particles, creating a bridge from the defect border to the centre of the defect, with a small amount of trabecular bone and some areas of mature bone; however, this was not statistically significant (Figs 5 and 6).

## Discussion

The objective of the present study was to develop a novel composite combining a porcine graft with collagen, evaluate its characteristics *in vitro* using New Zealand rabbit calvarial critical-size defects and determine its reliability as a bone graft biomaterial for new bone formation in future GBR treatments.

Based on the results, the porcine collagen graft showed rough particles of 500–1000 μm interconnected by collagen with a Ca/P ratio of 1.646, promoting cell viability and osteoblastic differentiation over 5 days. These characteristics were similar to those of the porcine graft and HA/β-TCP. In addition, these findings are in agreement with those of Maté Sánchez *et al*.^18^. They proposed that the surface roughness of biomaterials directly influences the possibility of creating a zone facilitating cell anchorage. Although a highly porous scaffold is preferred, as it favours bone cell adhesion and regeneration, it is achieved at the expense of mechanical strength and resistance. Even though the present study did not evaluate the material’s porosity, the porcine collagen particles showed roughness while they promoted cell viability in the MTT test. Maté Sánchez *et al*. also found that composite materials can be improved with the incorporation of collagen to achieve optimum physicobiological properties. The present study also found that collagen allows the internal replacement of the material with new bone in the rabbit calvarial defects over the 8 weeks, as demonstrated by the presence of new bone within the material and at its periphery^18^.

The porcine collagen composite in the present study had a ratio of 70:30 so that it mimics the natural human bone ratio, which is made up primarily of fine carbonated HA crystals (65%) and collagen matrices (23%). Additionally, the porcine collagen tried to imitate the organized 3D geometrical natural human bone structure, with the rationale of using many of the biological and mechanical properties present in natural human bone^21^. According to other authors, due to their effects on osteoblast gene expression, it is possible for high levels of Ca and P to stimulate osteogenesis^22^. Despite porcine collagen not having high levels of Ca and P due to the higher amount of collagen over the porcine graft, this composite presented a Ca/P ratio of 1.646, which is close to the stoichiometric value limit of 1.67 for pure calcium hydroxyapatite^23^.

New Zealand rabbit critical-size defect studies have established its validity as an experimental model for testing biomaterials used for bone replacement^24^. In the *in vivo* portion of the present study, not all of the biomaterials interfered with the normal bone repair process. Some authors argue that defects 6 mm in diameter are not critical-size defects, but the control defects in the present study were not able to reach complete closure; thus, the size of the defects are considered critical-size defects, which is in agreement with a previous study by the same authors^25^.

According to the micro-CT results, all the calvarial bone defects filled with the different bone grafts generated more new bone and cortical defect closure compared with the control. The porcine collagen composite generated statistically significantly more new bone compared with the porcine and HA/β-TCP grafts, which was attributed to the interconnectivity created by the collagen, which promotes angiogenesis^18^, creating the possibility of getting less new bone than what other studies report and despite not using barrier membranes to examine porcine collagen’s natural ability to form new bone.

It has been demonstrated that deproteinized bones not only lose their immune reactivity but also retain their osteoinduction and osteoconduction activities^26^. In the present study, the histology slides showed that both porcine graft and HA/β-TCP work as scaffolds from the defect’s borders toward the middle of the defect, without any immune reactivity, and are biocompatible, bioresorbable and osteoconductive; these results coincide with those of Guarnieri *et al*. who found that porcine xenograft is biocompatible and osteoconductive and does not interfere with the normal bone repair process^27^. For the last 3 decades, synthetic HA and β-TCP have been commercially available as bone graft substitute materials in the medical and dental fields, with good results in multiple studies^28–30^. The porcine collagen graft material shared the same characteristics with the porcine graft and HA/β-TCP and in histomorphometric analysis, demonstrated more new bone formation with a better critical-size defect space than those of the other graft materials. In another rabbit study performed on tibial defects, Calvo-Guirado *et al*. concluded that a collagenated porcine graft can be useful in daily practice depending on clinical needs. Even though the present study has limitations of being an *in vivo* and *in vitro* study with a short (8 weeks) follow-up period, the results support the conclusions of Calvo-Guirado *et al*. demonstrating that a porcine collagen composite has physicochemical properties that make it biocompatible and able to generate new bone formation through osteoconductivity.

## Materials and Methods

The materials tested in this study comprised a porcine bone graft, porcine collagen composite and HA/β-TCP. The procedure used to create the porcine graft in this study has been previously reported^25^. Briefly, the material was produced using high porosity porcine cortical long bones heated to 800 °C until particle sizes of 500–1000 μm were reached. The porcine collagen composite was a homogeneous plug consisting of purified porcine type I collagen fibers mixed with porcine bone graft with a weight ratio of 30:70. The mixture was poured into a mould and freeze-dried to yield porcine collagen composite in its final form. The HA/β-TCP was a biphasic ceramic material (MBCP™) consisting of 60% HA and 40% β-TCP with complete interconnected porosity of 70% which comprised macropores of >10 µm and micropores of <10 µm in a 2:1 ratio and sintered at temperatures >700 °C^14^.

### Scanning Electron Microscopy

Porcine collagen surfaces were sputter-coated with a 25-nm-thick layer of palladium gold using a sputtering apparatus (IB-2; Hitachi, Ltd, Tokyo, Japan), and porcine collagen composite surface morphology was observed using SEM (Model 2400; Hitachi, Ltd).

### Energy Dispersive Spectrometry

Elemental porcine collagen composite graft sample analysis was performed using SEM (Model 2400; Hitachi, Ltd), which is equipped with EDS.

### Cell Viability and Biocompatibility

Cell metabolic activity was evaluated according to succinic dehydrogenase activity using the MTT assay following the procedure described in the previous study,^30^. Briefly, in these experiments, porcine graft, porcine collagen composite and HA/β-TCP were added to Dulbecco’s Modified Eagle’s Medium solutions containing phosphate-buffered saline + polysialyltransferases in a 0.2 mg/mL ratio, while incubated in a humidified incubator at 37 °C. After 24 hours, MG-63 cells were seeded (1 × 10^4^ cells/mL) onto 24-well plates (Costar Corporation, Cambridge, MA, USA) and maintained in a humidified incubator with 5% CO_2_ and 95% air at 37 °C for 24 hours in the same type of medium previously described. The culture medium was aspirated thereafter, and all media solutions previously prepared with the graft materials were added to the cell culture wells, leaving just 1 well with the same untreated medium as a control group. After exposure for 5 days, the media solutions were aspirated at different times points at 1, 3 and 5 days, and MG-63 cells were cultured with dimethyl sulfoxide (DMSO) with 5% CO_2_ and 95% air at 37 °C and comprised the negative control group. Tetrazolium salt (MTT kit, Roche Applied Science, Mannheim, Germany) was added and metabolically reduced to coloured formazan by mitochondrial dehydrogenase in viable cells after incubating for 4 hours according to the manufacturer’s instructions. After solubilizing, the formazan dye was added to 500 μL DMSO for 5 minutes, and the optical density of the medium was determined using an ELISA reader (Model 2020, Anthos Labtec Instruments, Wals-Siezenheim, Austria) at a wavelength of 570 nm; the data were analysed by Student *t* test.

### Alkaline Phosphatase Assay

MG-63 cells were seeded (1 × 10^4^ cells/mL) in 500 µL of DMEM (medium A) in 4 plates of 24-well (Costar Corp.) and maintained in a humidified incubator with 5% CO_2_ and 95% air at 37 °C for 24 hours. Concurrently, porcine graft, porcine collagen composite and HA/β-TCP were mixed with DMEM (medium B) in a ratio of 0.2 mg/mL and kept at 27 °C for 24 hours. Later, medium A was removed and substituted with medium B. Samples were taken after 3 freeze-thaw cycles of cell membrane rupture with 0.5% Triton X-100. Each plate was used immediately after the substitution and 1, 3 and 5 days later. Samples were preserved at −80 °C for 24 hours.

Using a 96-well (Costar Corp.), a total protein assay was performed by mixing 100 µL of the sample with 100 µL of solution made with 5 mL of Bio-Rad protein assay reagent plus 20 mL Deionized water. In order to perform the ALP assay, 9 mL of solution integrated with 8 mL alkaline buffer solution 1.5 M plus 4 mL DII water was mixed with 16 µmole of MgCl_2_ (SIGMA 104–40), and 100 µL of the solution were set within 100 µL of previously obtained sample in a 96-well (Costar Corp.). Absorbance in both tests was measured at 405 nm with 620 nm as a reference using ELISA reader (Plate Chameleon Multilabel Detection Platform; HIDEX, Finland) and absorbance data were analysed.

### Surgical Procedure

**Figure 7:**
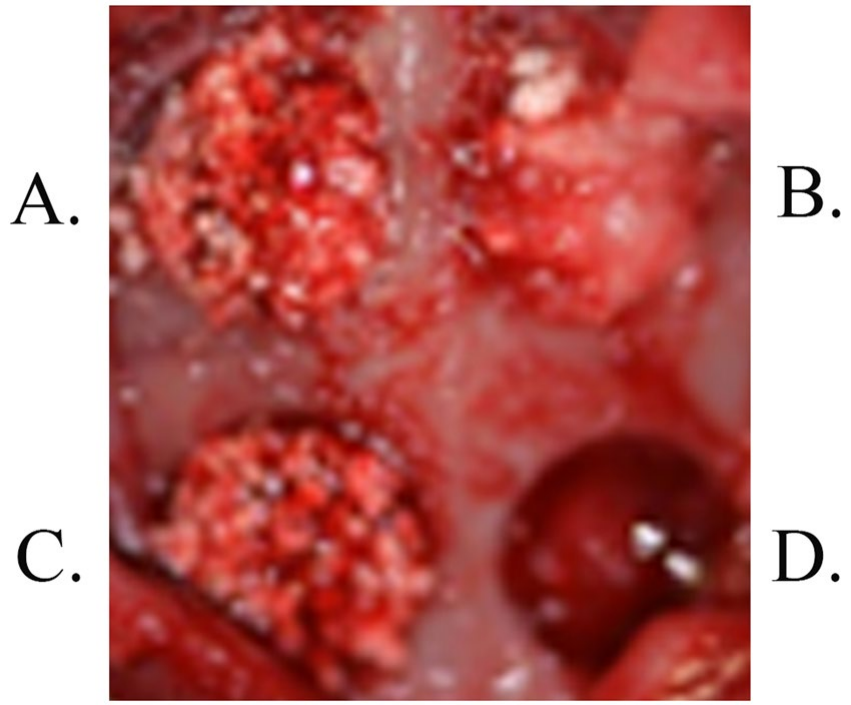
Rabbit calvarial cortical defects. Surgical procedure: (**A**) Porcine graft, (**B**) Porcine collagen composite, (**C**) HA/β-TCP and (**D**) Control.

Surgical procedures were performed in 20 adult male New Zealand white rabbits with a mean age of 12 weeks and a mean weight of 2.1 kg. All experimental protocols were approved by the animal ethics committee of Taipei Medical University, and all experiments were performed in accordance with relevant guidelines and regulations. The animals were housed in cages at 19 °C and 55% humidity at the Taipei Medical University Laboratory Animal Center and fed standard rabbit chow and water *ad libitum*. Anaesthesia was administered using an intramuscular injection of Zoletil 50 (50 mg/mL) at 15 mg/kg into the gluteal region, and surgery was performed in animals after 10 minutes of sedation. The calvarial region was then shaved, draped and sterilized using iodine, and 1.8 mL of 2% lidocaine with epinephrine 1/100,000 was injected as a haemostatic. Subsequently, a 2-cm longitudinal midline vertical skin incision was made, the periosteum was retracted to expose the calvarial bones and 4 critical calvarial defects of 6-mm diameter and 3-mm depth were prepared using a trephine bur (3I Implant Innovation, Palm Beach Gardens, FL, USA) bilaterally in the parietal and frontal bones of each rabbit. Each defect was considered a critical size defect that would not heal during the animal’s lifetime. The upper left defect was filled with porcine graft, the upper right with the porcine collagen composite, the lower left with HA/β-TCP (MBCP; Biomatlante biologics solutions) and the lower right defects were unfilled (controls). The rabbits were kept in cages under surveillance for the first 24 hours and then examined every 3 days for 2 weeks and weekly thereafter (Fig. 7). The animals were grouped for sacrifice at 2, 4, 6 and 8 weeks after surgery. Euthanasia was performed by CO_2_ asphyxiation 10 minutes after intramuscular injection of Zoletil 50 (50 mg/mL) at 15 mg/kg into the gluteal region. Subsequently, a section was made between the 4 critical defects. Sample blocks were prepared in formalin and micro-CT scanning analyses were performed within 2 weeks using SkyScan 1076 (Antwerp, Belgium)^25^.

### Micro-CT Scan

After obtaining the micro-CT images, coronal images of the defects were saved in the database, and 3D morphological analyses were performed on 20 samples. Morphometric parameters were calculated on individual binarized cross-sectional images, and 2D morphometric parameters were determined slice-by-slice and integrated across multiple slices for a complete 3D analysis. After integration of whole volume of interest (VOI), percentages of VOI occupied by binarized solid objects were calculated, and this parameter comprised percentages of the total volume (TV) of VOI and the total binarized volume (BV) of objects within the VOI. Moreover, to measure the new bone formation, binary selections of samples from the morphometric analyses were made according to grayscale density between units of 20 and 80, respecting the 6-mm diameter limits of the calvarial defects.

### Histology and Histomorphometric Analysis

Bone specimens were decalcified in formalin. Decalcification times, specimen thicknesses, temperature, decalcification solution freshness and block samples’ decalcification conditions were recorded. Specimens were washed in sterile distilled water for several hours following decalcification,. Subsequently, bone tissues were dehydrated in alcohol and embedded in liquid paraffin. Two slices with 7 μm-thickness were taken from the middle of each bone specimen, and hematoxylin and eosin staining was performed on all paraffin-embedded tissues. Histological slides were performed on the sagittal view of the defect using a Leica/Aperio ScanScope System, and histologic image analysis was performed at 20× magnification, whereas morphometric analysis was done by sampling mean new bone formation from 3 regions of interest, each with a size of 1.5 × 1.5 mm, 2 on the calvarial defect borders and a third in the middle of the defect. ImageJ software (National Institutes of Health; Bethesda, MD, USA) was used to measure new bone formation.

### Statistical Analysis

Descriptive statistics including mean values and standard deviations were used for all tests. The Jarque–Bera test was used to test normality. Data analysis and comparisons in all tests were performed using Student *t* test (Microsoft Excel, Office 2016). Comparisons between the 2 groups with *P*-values < 0.05 were considered statistically significant differences.

## Conclusion

With the limitations of the present study, porcine collagen showed good physicochemical properties, was biocompatible and generated new bone formation through osteoconductivity, proving to be a reliable bone graft biomaterial option for future GBR treatments.
